# Vancomycin Flushing Syndrome: A Case Report

**DOI:** 10.7759/cureus.58487

**Published:** 2024-04-17

**Authors:** Shitij Shrivastava, Shashwat Shrivastava

**Affiliations:** 1 Internal Medicine, BronxCare Health System, New York City, USA; 2 Medicine, California Institute of Behavioral Neurosciences & Psychology, Fairfield, USA; 3 Cardiothoracic Surgery, New York University, New York City, USA

**Keywords:** hypersensitivity, vancomycin flushing syndrome, vancomycin, pharmacology, clinical infectious medicine

## Abstract

Vancomycin is a bactericidal antibiotic used for various infections but can cause hypersensitivity reactions, including vancomycin flushing syndrome (VFS) and anaphylaxis. VFS, previously known as red man syndrome, is a pseudoallergic reaction characterized by flushing, erythema, and pruritus. We present a case of VFS in a female patient with recurrent Methicillin-resistant Staphylococcus aureus (MRSA) infections receiving vancomycin for back abscesses. Following the second dose, she developed a pruritic rash on her face, neck, and torso, which resolved with treatment. The differential diagnosis included hydromorphone allergy, ruled out due to previous tolerance. Anaphylaxis was unlikely due to the absence of respiratory distress, hypotension, or angioedema. Management involved discontinuing vancomycin, administering corticosteroids and antihistamines, and monitoring for anaphylaxis. The patient was transferred for surgical intervention and alternative antibiotic therapy. This case highlights the importance of recognizing and managing VFS, the significance of differential diagnoses, and the need for enhanced documentation and clinical support in managing vancomycin hypersensitivity reactions.

## Introduction

Vancomycin is a bactericidal antibiotic used for a wide variety of infections. It can cause two types of hypersensitivity reactions, vancomycin flushing syndrome and anaphylaxis. VFS was previously referred to as red man syndrome. VFS has been reported in 3.7% to 47% of infected patients [1]. It is a pseudoallergic reaction characterized by flushing, erythema, and pruritus, affecting the upper body, neck, and face more than the lower body. The reaction or the severity of the VFS can vary. Some patients will not have this reaction until after they have received multiple doses or have had a slow infusion. Delayed reactions after 90 to 120 minutes have also been observed. Symptoms may occur as soon as four minutes after initiating the first dose until up to seven days after dose completion [2]. The diagnosis is made clinically. One of the mechanisms involved includes the release of histamines, which cause the classic flushing associated with VFS and require the presence of preformed antibodies [3]. In this article, we describe a case of VFS in a female patient who was receiving vancomycin for back abscesses and had a history of recurrent methicillin-resistant Staphylococcus aureus (MRSA) infections.

## Case presentation

**Figure 1:**
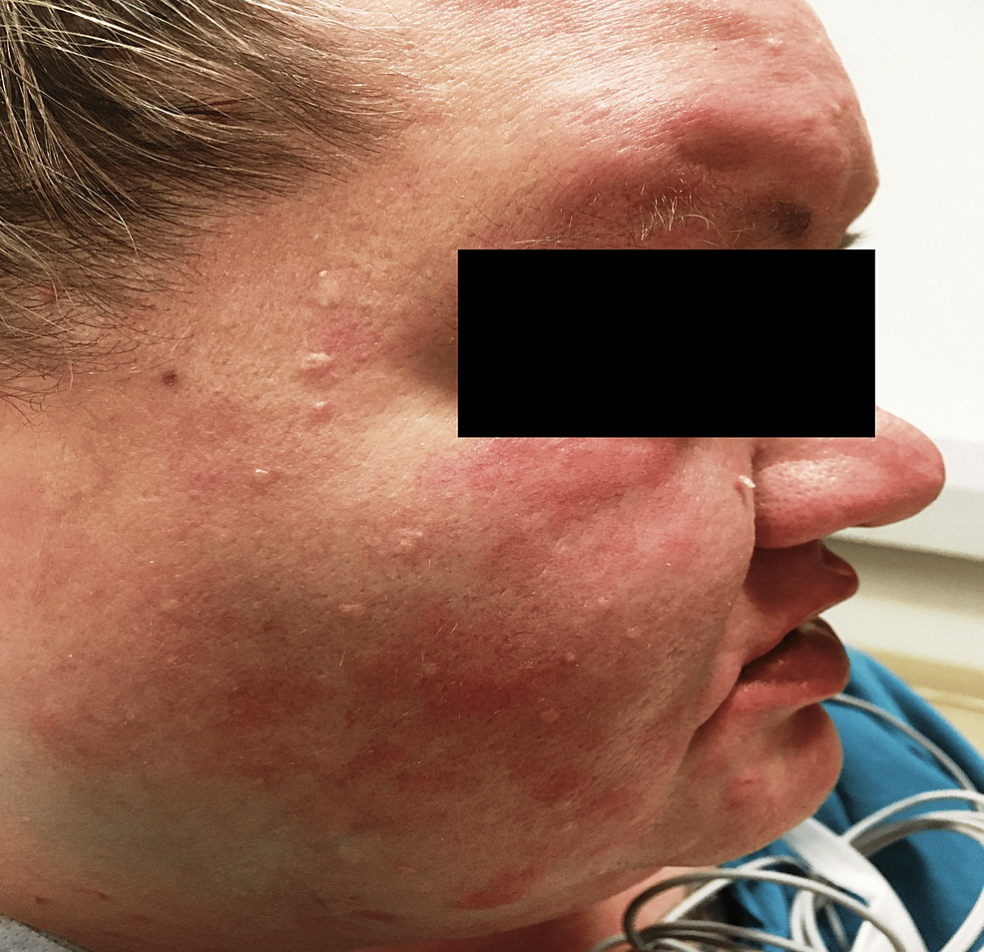
Facial erythema and raised urticarial eruption most notable over the lower eyelid, cheek, perioral region, and forehead

**Figure 2:**
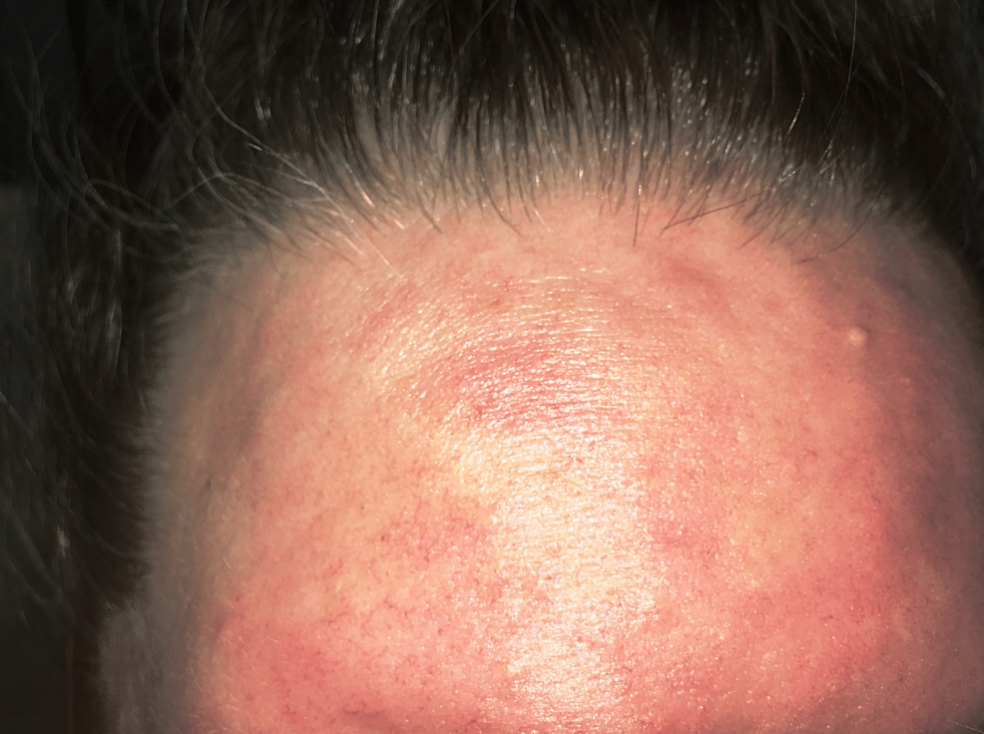
Forehead erythema, urticaria, and flushing

A 44-year-old female with a history of recurrent MRSA infections and psoriasis presented to the Emergency Room with three back abscesses getting progressively painful for five days. Laboratory data showed lactic acid of 2.5 mg/dl, white blood cell count of 12,100 K/uL, and segmented neutrophils at 86%. Vital signs were normal. A physical exam showed three abscesses over the back, with associated fluctuance. Due to the history of recurrent MRSA infections and abscesses, she was admitted to the medical floor for intravenous antibiotics and abscess drainage. She was started on 0.1% triamcinolone acetonide cream, 0.5 mg/ml hydromorphone as needed, and, vancomycin (1.5 g infusion/150 minutes in 500 ml of normal saline every 12 hours). Overnight, the patient developed a raised, erythematous, pruritic, urticarial rash on her face, neck, and torso, 50 minutes into her second dose of vancomycin. The patient denied any similar episodes in the past but she did have a similar reaction to ceftriaxone and morphine. The patient had also received hydromorphone 90 minutes before this episode. Vancomycin and hydromorphone were immediately discontinued and vitals were checked. The patient had tachycardia of 102 beats per minute but was normotensive. Two doses of 40 mg intravenous methylprednisolone, a single dose of 50 mg intravenous diphenhydramine, and 20 mg of intravenous famotidine were administered. The rash and itching resolved shortly afterward. The patient was transferred to a tertiary care center due to the need for surgical intervention and the risk of developing anaphylaxis to the next antibiotic. A complete blood count test was ordered shortly after the hypersensitivity reaction, which showed an eosinophil differential of 5%. Figure 1and Figure2* *depict the facial and forehead erythema and raised urticarial eruption seen in our patient.

## Discussion

Vancomycin works by inhibiting the synthesis of bacterial cell walls. It does this by binding to the D-alanyl-D-alanine terminus of cell wall precursor units, preventing their incorporation into the peptidoglycan matrix, which is essential for bacterial cell wall formation. The differential diagnoses include hydromorphone allergy and vancomycin allergy. The patient had tolerated hydromorphone and vancomycin well during the previous visits so both allergies were ruled out. The patient didn’t have respiratory distress, hypotension, or angioedema, hence anaphylaxis was determined to be unlikely and was ruled out as well. Opiates and muscle relaxants can also trigger histamine release [4]. Therefore, VFS is worse in patients who not only have received vancomycin but also muscle relaxants or opioid analgesics. Following VFS, the medication list should be reviewed to determine if other predisposing medications like opiates need to be discontinued before restarting the infusion. Each intravenous dose of vancomycin should be administered over at least a 60-minute interval [5] to minimize infusion-related adverse effects. Longer infusion times should be used in patients receiving doses considerably larger than 1 gram of vancomycin, with some literature suggesting better tolerance when given more frequently in smaller doses [4,6]. Vancomycin must be immediately discontinued once VFS is suspected. Pretreatment with antihistamines such as hydroxyzine or diphenhydramine can offer protection against VFS [4,7]. One must consider vancomycin desensitization for severe reactions that do not respond to standard measures in cases of anaphylactic reactions to vancomycin, and when switching to another antibiotic is not possible and vancomycin is required. Rapid desensitization is preferred, as it enables re-dosing of vancomycin within 24 hours [1]. Wong et al. reported that rapid continuous intravenous administration of small doses of vancomycin with progressively increasing dosage is an alternative option in patients who do not respond to slowing of the infusion rate [7]. Vancomycin flushing syndrome has also been reported in patients receiving intraperitoneal vancomycin, oral vancomycin, and vancomycin powder on surgical wounds for infection prophylaxis [4,8,9]. Randomized trials regarding treatment for VIR have not been conducted and treatment is focused on the severity of reactions. That being said, antihistamines are the mainstay of treatment [5].

The term “Red Man’s Syndrome” was first used by Garrelts and Peteri in 1985 in the New England Journal of Medicine [10]. Vancomycin was discovered in 1953 by Edmund Kornfeld from cultures of Amycolatopsis orientalis (formerly Streptomyces orientalis) obtained from the jungles of Borneo. In the 1960s and 1970s, its utilization experienced a significant decrease, primarily because of the elevated occurrence of adverse reactions and the emergence of semisynthetic penicillins possessing a broader spectrum of therapeutic effectiveness in the medical toolkit [11]. It was first used clinically in the 1950s, primarily for the treatment of penicillin-resistant Staphylococcus aureus infections. Vancomycin is primarily used to treat serious infections caused by Gram-positive bacteria, including MRSA, methicillin-resistant Staphylococcus epidermidis, and Enterococcus species. The first isolates of MRSA were described in 1961 by Patricia Jevons [12]. Vancomycin continues to be a vital antibiotic in the treatment of serious gram-positive infections, including those caused by MRSA. Its use and efficacy are discussed in numerous, contemporary medical literature such as in this study by Rybak et al. [13]. Recently, significant literature has been published to abort the use of the term “red man syndrome” [14-16], as it is truly considered stereotypical, stigmatizing, and bias-inducing toward Native Americans. Native Americans, also known as American Indians or First Americans, are the indigenous peoples of the United States, including Hawaii and other territories of the United States, and are at other times limited to the mainland. They are indigenous tribes that were in the Americas before the arrival of Europeans in the 15th century. Since then, the Infectious Diseases Society of America (IDSA), its HIV Medicine Association (HIVMA), the Society for Healthcare Epidemiology of America (SHEA), the Pediatric Infectious Diseases Society (PIDS), and the Society of Infectious Diseases Pharmacists (SIDP) issued a statement in September 2021, which supported the abolishment of the term “red man syndrome” and it was changed to “vancomycin infusion reaction”.

Arrango et al. conducted a study aimed at examining the epidemiology of hypersensitivity reactions to vancomycin using electronic health record allergy data. Conducted over 2017-2019 in two healthcare systems in the United States, it found that among 4,490,618 patients, 14,426 (0.3%) had a vancomycin drug allergy label. Of the 18,761 documented reactions, 12.0% were in free-text. Rash (32%) and red man syndrome (RMS, 16%) were common reactions, with anaphylaxis coded in 6% of cases, although true IgE-mediated reactions are rare. VFS documentation was more common in males and less common in Blacks. Enhanced documentation of vancomycin reactions and targeted clinical decision support are necessary to enhance inpatient care and advance research on vancomycin allergy epidemiology [17].

## Conclusions

This case emphasizes the importance of recognizing and managing vancomycin flushing syndrome (VFS), a pseudoallergic reaction that can mimic severe allergic responses. Prompt recognition and appropriate management are crucial, as VFS can be mistaken for true allergies, leading to unnecessary avoidance of an effective antibiotic. The evolving terminology from "red man syndrome" to "vancomycin infusion reaction" reflects a commitment to respectful language in medical practice. Enhanced documentation and clinical decision support systems are vital for managing vancomycin allergies and improving patient care. Further research is needed to understand the epidemiology and optimal management of vancomycin hypersensitivity reactions.
